# Genetic responses of inbred chicken lines illustrate importance of eIF2 family and immune-related genes in resistance to Newcastle disease virus

**DOI:** 10.1038/s41598-020-63074-9

**Published:** 2020-04-09

**Authors:** Ana Paula Del Vesco, Michael G. Kaiser, Melissa S. Monson, Huaijun Zhou, Susan J. Lamont

**Affiliations:** 1 Department of Animal Science, Iowa State University, Ames, IA USA; 2 Department of Animal Science, Universidade Federal de Sergipe, São Cristóvão, SE Brazil; 3 Department of Animal Science, University of California, Davis, CA USA

**Keywords:** Agricultural genetics, Viral infection

## Abstract

Newcastle disease virus (NDV) replication depends on the translation machinery of the host cell; therefore, the eukaryotic translation initiation factor 2 (eIF2) gene family is a likely candidate for control of viral replication. We hypothesized that differential expression of host genes related to translation and innate immune response could contribute to differential resistance to NDV in inbred Fayoumi and Leghorn lines. The expression of twenty-one genes related to the interferon signaling pathway and the eIF2 family was evaluated at two- and six-days post infection (dpi) in the spleen from both lines, either challenged by NDV or nonchallenged. Higher expression of *OASL* in NDV challenged versus nonchallenged spleen was observed in Leghorns at 2 dpi. Lower expression of *EIF2B5* was found in NDV challenged than nonchallenged Fayoumis and Leghorns at 2 dpi. At 2 dpi, NDV challenged Fayoumis had lower expression of *EIF2B5* and *EIF2S3* than NDV challenged Leghorns. At 6 dpi, NDV challenged Fayoumis had lower expression of *EIF2S3* and *EIF2B4* than NDV challenged Leghorns. The genetic line differences in expression of eIF2-related genes may contribute to their differential resistance to NDV and also to understanding the interaction between protein synthesis shut-off and virus control in chickens.

## Introduction

Newcastle disease virus (NDV) outbreaks have been reported in several countries in the last few decades and continue to cause economic losses around the world^1^. Newcastle disease virus can cause different symptoms depending on strain pathogenicity, concurrent diseases, avian species, and genetic resistance to the pathogen. Symptoms of NDV range from morbidity and respiratory signs associated with lentogenic strains to high mortality caused by velogenic strains^2^. Birds infected with NDV exhibit increased expression of cytokines^3^ and genes related to antiviral action of interferons (IFNs) and chemokines^4^.

As an sRNA virus belonging to the Paramyxoviridae family, NDV produces a double-stranded molecule (dsRNA) during its replication process^5^. To contain the virus and prevent it from spreading, type I interferons bind to their receptors and signal to downstream molecules to stimulate the transcription of several interferon-stimulated genes (ISGs)^6^. In chickens, some of the ISGs are 2′-5′-oligoadenylate synthetase (OAS) and protein kinase R (PKR). These ISGs act at different stages of the viral replication cycle and can be upregulated by NDV infection^7,8^; OAS turns on degradation of viral RNA, and PKR acts to contain viral replication^9^.

Newcastle disease virus replication depends on the translation machinery of the host cell and thus it is subject to the control mechanisms modulated by host translation factors^10^. The first stage of protein translation is the most regulated phase and eukaryotic initiation factor (eIF) family genes play important roles in this regulation^11^. Eukaryotic initiation factor 2 (eIF2) is a protein complex with three subunits, eIF2α, eIF2β and eIF2γ^12^ encoded by *EIFS1*, *EIFS2* and *EIFS3* genes, respectively, while eIF2B has five subunits, eIF2Bα to eIF2Bε^13^ encoded by *EIF2B1* to *EIF2B5* genes, respectively. The beginning of translation depends on the formation of the preinitiation complex composed by the 40 s ribosomal subunit, eIF3, eIF1A and the ternary complex: eIF2:GTP:tRNA^Met^. Under normal conditions eIF2:GDP binds to a guanine nucleotide exchange factor, eIF2B, which makes the exchange of GDP for GTP^14^. However, phosphorylation of the eIF2α subunit of eIF2 by one of the eIF2 kinases, such as the protein kinase R (PKR, encoded by *EIF2AK2* gene) or protein kinase R-like endoplasmic reticulum kinase (PERK, *EIF2AK3* gene), inhibits the initiation of translation and causes translation shut-off by preventing the conversion of eIF2-GDP into eIF2-GTP by eIF2B^15^. Factors such as stress in the endoplasmic reticulum^16^ and the presence of viral RNA^17^ may stimulate the phosphorylation of eIF2α by PERK and PKR, respectively. The dsRNA generated during NDV infection stimulates PKR activity, and thus, eIF2α phosphorylation, which is followed by antiviral action in infected cells^18^.

A role for the eIF2 signaling pathway has recently been reported in studies of the transcriptome of two different inbred chicken lines, Fayoumi and Leghorn, when challenged by NDV^19–21^. These two lines have been used to evaluate the mechanisms of genetic response to several different pathogens^22–24^. During NDV infection, the Fayoumis had faster viral clearance than the Leghorns from 2- to 6-days post infection (dpi) and higher serum antibody level at 10 dpi compared to the Leghorns, and thus the Fayoumi are considered to be relatively more resistant to NDV than the Leghorns^20,25^. Several ISGs were upregulated in NDV challenged chickens from the two lines; therefore, we hypothesized that some pathways specifically activated in challenged Fayoumis might contribute to the greater relative resistance to NDV observed in the Fayoumi chickens. Pathway analysis of transcriptome data from trachea^20^ and spleen^21^ tissues have shown that eIF2 signaling may be one of the pathways related to higher resistance to NDV.

We hypothesize that differential expression of genes related to the host translation machinery and immune response play a role in containing NDV replication and that genetic control of gene expression differences may contribute to relative resistance to NDV infection. To assess our hypothesis, the expression of genes related to the interferon-signaling pathway and the eIF2 family were evaluated in the spleen of Fayoumi and Leghorn chickens after NDV challenge. We report for the first time an association between the expression of the eIF2 and eIF2B subunits and NDV infection in chickens.

## Results

### NDV challenge effect

**Figure 1:**
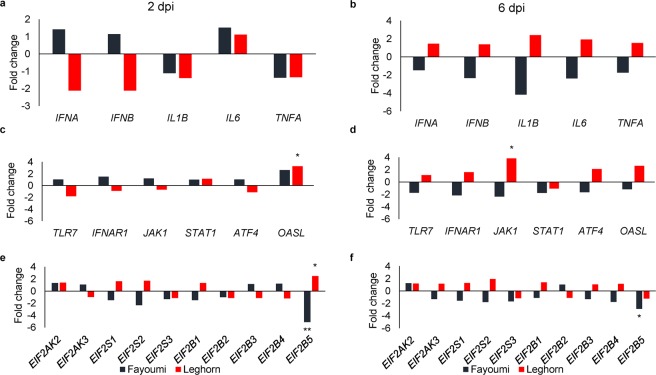
NDV challenge effect on the expression of (**a**,**b**) cytokines, (**c**,**d**) receptors and signaling molecules, and (**e**,**f**) eIF2 family-related genes in the spleen of chickens at 2- and 6-days post infection (dpi). The bars represent the fold change between the NDV challenged and nonchallenged within genetic line: Fayoumis (black bars) and Leghorns (red bars). *FDR < 0.1, **FDR < 0.05.

To evaluate the effect of NDV challenge, host responses were assessed by comparing expression of 21 genes between NDV challenged and nonchallenged spleens within the Fayoumi and the Leghorn chicken lines at 2 and 6 dpi (Table 1; Fig. 1). At 6 dpi, Janus kinase 1 (*JAK1*) expression was higher in the spleen of NDV challenged than nonchallenged Leghorns (*FDR* = 0.06; Fig. 1d). NDV challenge effect was also observed in the expression of 2′-5′-oligoadenylate synthetase like (*OASL*), an ISG related to dsRNA degradation; higher expression of this gene in NDV challenged versus nonchallenged spleens was found in Leghorns at 2 dpi (*FDR* = 0.08; Fig. 1c). The NDV challenged Fayoumis had lower expression of the *EIF2B5* gene than nonchallenged Fayoumis at 2 dpi (*FDR* = 0.02; Fig. 1e) and 6 dpi (*FDR* = 0.07; Fig. 1f). Expression patterns also showed lower expression of the *EIF2B5* gene in NDV challenged than nonchallenged Leghorns at 2 dpi (*FDR* = 0.09; Fig. 1e).

**Table 1:** Gene expression in the spleen of Fayoumi and Leghorn chickens NDV challenged or nonchallenged at 2 and 6-days post infection (dpi).

Major category		2 dpi					6 dpi					
		Fayoumi		Leghorn			Fayoumi			Leghorn		
		NDV^2^	Non^3^	NDV^2^		Non^3^	NDV^2^	Non^3^		NDV^2^	Non^3^	
Receptors	*TLR7*	18.83 ± 0.3	18.81 ± 0.5		18.23 ± 0.3	19.09 ± 0.5	17.75 ± 0.8		18.55 ± 0.8	19.26 ± 0.7		19.09 ± 0.8
	*IFNAR1*	16.59 ± 0.6	16.01 ± 0.6		15.65 ± 0.6	15.83 ± 0.6	14.94 ± 0.6		16.06 ± 0.6	16.14 ± 0.5		15.46 ± 0.6
Cytokines	*IFNA*	21.16 ± 0.7	20.66 ± 0.8		19.07 ± 0.5	20.15 ± 0.8	19.47 ± 1.0		20.03 ± 1.0	21.18 ± 0.5		20.63 ± 1.0
	*IFNB*	15.06 ± 0.6	14.87 ± 0.7		12.93 ± 0.6†	14.01 ± 0.7	13.66 ± 0.7		14.88 ± 0.7	15.46 ± 0.6†		15.00 ± 0.7
	*IL1B*	13.80 ± 0.5	13.95 ± 0.6		13.70 ± 0.5	14.17 ± 0.6	12.81 ± 0.8		14.87 ± 0.8	15.42 ± 0.7		14.16 ± 0.8
	*IL6*	12.48 ± 0.4	11.88 ± 0.5		12.10 ± 0.4	11.94 ± 0.5	11.51 ± 0.2		12.76 ± 0.2	13.21 ± 0.6		12.27 ± 0.7
	*TNFA*	12.15 ± 0.15	12.60 ± 0.6		12.10 ± 0.5	12.52 ± 0.6	10.76 ± 0.6		11.56 ± 0.6	12.66 ± 0.5		12.04 ± 0.6
Signaling molecules	*JAK1*	18.94 ± 0.4†	18.68 ± 0.5		18.32 ± 0.4	18.85 ± 0.5†	17.49 ± 0.5 A†		18.72 ± 0.5 A	19.09 ± 0.4Ba		17.15 ± 0.5Bb†
	*STAT1*	13.78 ± 0.3	13.78 ± 0.4		13.91 ± 0.3	13.70 ± 0.4	12.27 ± 0.5		13.09 ± 0.5	13.65 ± 0.4		13.75 ± 0.5
	*ATF4*	16.35 ± 0.6	16.31 ± 0.7		15.84 ± 0.6	16.01 ± 0.7	15.22 ± 0.5		15.95 ± 0.5	16.87 ± 0.4		15.79 ± 0.5
ISG^1^	*OASL*	16.56 ± 0.4†	15.17 ± 0.4		17.30 ± 0.4a	15.59 ± 0.4b	14.81 ± 0.5†		15.03 ± 0.5	15.89 ± 0.4		14.51 ± 0.5
eIF2 family	*EIF2AK2*	17.44 ± 0.4	17.05 ± 0.4		17.61 ± 0.4	17.15 ± 0.4	16.47 ± 0.4		16.14 ± 0.4	17.08 ± 0.3		16.81 ± 0.4
	*EIF2AK3*	12.65 ± 0.4	12.56 ± 0.4		12.37 ± 0.3	12.41 ± 0.4	11.70 ± 0.3		12.08 ± 0.3	13.00 ± 0.2		12.76 ± 0.3
	*EIF2S1*	17.60 ± 0.3	18.19 ± 0.2		18.14 ± 0.3	17.45 ± 0.2	16.80 ± 0.5		17.43 ± 0.4	18.83 ± 0.4		18.46 ± 1.1
	*EIF2S2*	18.98 ± 0.4	20.19 ± 0.4		19.77 ± 0.4	19.00 ± 0.4	18.59 ± 0.7		19.42 ± 0.4	20.65 ± 0.6		19.70 ± 0.7
	*EIF2S3*	14.37 ± 0.3 **A**	14.77 ± 0.4 **A**		18.60 ± 0.3**B**	18.80 ± 0.4**B**	13.58 ± 0.3 **A**		14.32 ± 0.3 **A**	18.87 ± 0.3**B**		19.08 ± 0.5**B**
	*EIF2B1*	13.11 ± 0.4	13.68 ± 0.4		13.10 ± 0.3	12.71 ± 0.4	12.86 ± 0.6		13.02 ± 0.4	13.77 ± 0.6		13.30 ± 0.4
	*EIF2B2*	17.62 ± 0.4	17.64 ± 0.5		16.92 ± 0.4	17.11 ± 0.4	17.09 ± 0.3		17.05 ± 0.4	17.33 ± 0.3		17.48 ± 0.4
	*EIF2B3*	14.93 ± 0.4	14.72 ± 0.6		14.16 ± 0.4	14.34 ± 0.3	13.91 ± 0.3		14.29 ± 0.4	14.77 ± 0.3		14.68 ± 0.5
	*EIF2B4*	12.08 ± 0.4*	11.79 ± 0.5		11.27 ± 0.4	11.05 ± 0.4	10.25 ± 0.3 A*		11.06 ± 0.3	11.80 ± 0.3B		11.61 ± 0.2
	*EIF2B5*	10.11 ± 0.4**Aa**	12.46 ± 0.7A**b**		12.11 ± 0.4**B**a*	10.81 ± 0.7Bb	10.82 ± 0.3a		12.35 ± 0.3Ab	10.45 ± 0.3*		10.72 ± 0.6B

Pairwise comparisons between groups were based on a linear model and tested effects of line, challenge status, dpi. Significant line differences are shown as bold capitalized letters (FDR < 0.05) and trends as capitalized letters (FDR < 0.1). Significant differences due to challenge status are indicated by bold small letters (FDR < 0.05) and trends by small letters (FDR < 0.1). Significant differences across dpi are illustrated with *(FDR < 0.05) and trends with ^†^(FDR < 0.1).

^1^ISG, Interferon stimulated genes. ^2^NDV, NDV challenged. ^3^Non, nonchallenged.

### Line effect

**Figure 2:**
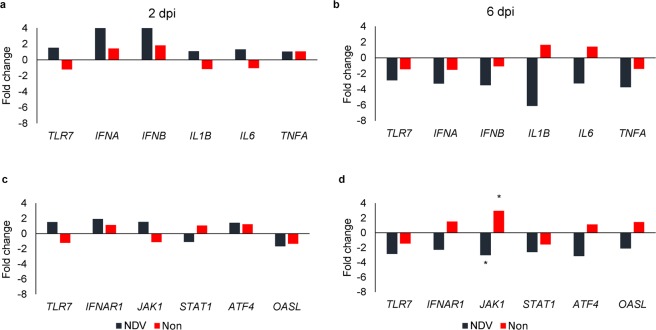
Genetic line effect on the expression of (**a**,**b**) cytokines, and (**c**,**d**) receptors and signaling molecules in the spleens of chickens at 2- and 6-days post infection (dpi). The bars represent the fold change between the Fayoumis and the Leghorns within treatment: NDV challenged (black bars) or nonchallenged chickens (red bars). *FDR < 0.1.

To further evaluate differences in the response of each line to NDV challenge, we compared the gene expression of receptors, cytokines and signaling molecules related to the early-antiviral activity between Fayoumis and Leghorns within challenge status and dpi (Table 1; Fig. 2). For these genes, line effect was only found for *JAK1* expression where Leghorns had higher expression of this gene at 6 dpi in both challenge statuses (*FDR* = 0.07; Fig. 2d).

**Figure 3:**
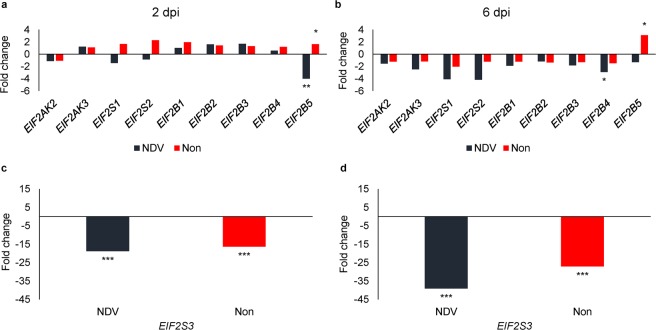
Genetic line effect on the expression of (**a**,**b**) eIF2 family-related genes and *EIF2S3* gene (**c**,**d**) in the spleen of chickens at 2 and 6-days post infection (dpi). The bars represent the fold change between the Fayoumis and the Leghorns within NDV challenged (black) and nonchallenged chickens (red bars). *FDR < 0.1, **FDR < 0.05, ***FDR < 0.001.

Because Fayoumis had faster NDV clearance than the Leghorns as previously reported^20^, we also evaluated the differences between lines in the expression of eIF2 family-related genes to better understand how NDV replication may be delayed by host protein synthesis shut-off (Table 1; Fig. 3). Within nonchallenged chickens, effect of chicken genetic line was found on the expression of two eIF2 genes: Fayoumis had higher expression of *EIF2B5* (2 and 6 dpi; both *FDR* = 0.06; Fig. 3a,b), and highly significant lower expression of *EIF2S3* (2 and 6 dpi; both *FDR* < 0.001; Fig. 3c,d) than the Leghorn chickens. Comparing genetic lines within NDV challenged chickens, the Fayoumis again had highly significant lower expression of *EIF2S3* (2 and 6 dpi; both *FDR* < 0.001; Fig. 3c,d) than the Leghorns. NDV challenged Fayoumis also had significantly lower expression of *EIF2B5* compared to the Leghorn chickens, but only at 2 dpi (*FDR* = 0.02; Fig. 3a). The highest fold changes were observed in expression of the *EIF2S3* gene. The lower expression of eIF2 family genes in NDV challenged Fayoumis may illustrate a mechanism of resistance, in that NDV propagation may be delayed in Fayoumis compared to Leghorn due to a reduction in the host protein synthesis machinery. Different subunits of eIF2 and eIF2B with different functions can be regulated to help the innate immune response contain NDV replication.

### Day post infection effect

**Figure 4:**
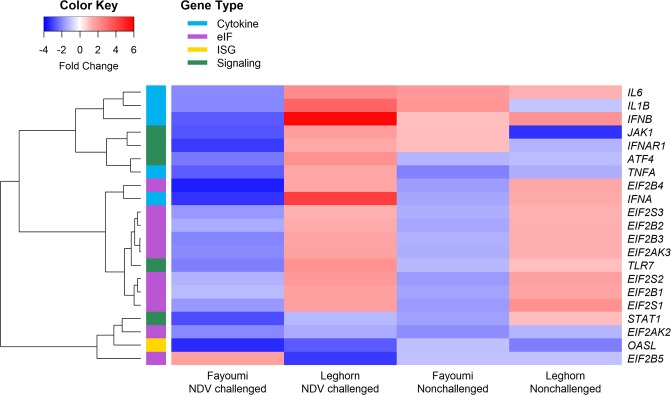
Heat map showing the fold change between 6- and 2-days post infection within line and challenge status in the spleen. See Table 1 and Supplementary Table S1 for significance.

The heat map (Fig. 4) shows comparisons between 6 and 2 dpi within line and challenge statuses. NDV challenged Fayoumis had higher expression of the *JAK1* (*FDR* = 0.07), *OASL* (*FDR* = 0.08) and *EIF2B4* (*FDR* = 0.04) genes at 2 than at 6 dpi (Table 1; Fig. 4). Within NDV challenged Leghorns, higher expression of *EIF2B5* (*FDR* = 0.04) and lower expression of *IFNB* (*FDR* = 0.09) were observed at 2 than at 6 dpi. The patterns of gene expression for the NDV challenged Fayoumis and Leghorns can be helpful to understand how each line can repress the replication of NDV at early stages of infection.

## Discussion

The severity of infection with NDV is determined by many different factors such as tissue tropism, efficiency of replication, concurrent diseases, and host resistance to the virus^26^. Some of the NDV response traits are known to have a component of genetic control^27^. Gene expression changes in the immune response to the NDV challenge in two highly inbred lines and their sublines, the Fayoumi (M5.1 and M15.2) and the Leghorn chickens (Ghs6 and Ghs13), have shown that the Major Histocompatibility Complex (MHC) can contribute to the genetic resistance against NDV^28^. Evaluation of the transcriptome of these two genetically distinct lines also suggests that NDV infection may cause upregulation of different genes in a line-dependent manner^19^. These results were also observed in the spleen, where few interferon stimulated genes were upregulated by NDV challenge^21^.

In the current study, the observed time effect on the expression of *IFNB* may help to explain the different rates of viral clearance between lines, as the Fayoumi had lower viral load than the Leghorn at 6 dpi^19,20^. Recognition of viral RNA by TLR7 triggers signaling for cytokines with antiviral activity to be upregulated^29^. Infection with NDV causes a strong immune response as showed by the upregulation of different cytokines and chemokines^4^. Type I interferon production is also induced by NDV infection^30^ and favors an effective host response. Interferon α and β bind to a common receptor comprising IFN-α receptor 1 (IFNAR1) and IFNAR2 subunits and signal through the JAK-STAT pathway to induce the transcription of several ISGs related to the antiviral response^31^. Although, IFNα and β play an important role against viral infection, each one may signal through different ISGs and may have different antiviral activities^32^. IFNβ may be mainly committed to signaling and immune modulation, while IFNα could have the strongest antiviral activity against vesicular stomatitis virus, avian influenza virus, and NDV infection in chickens^32^. Because interferons act in the first line of defense against viruses, the expression pattern for *IFNB* after infection, i.e. higher expression in Leghorn at the later time, could indicate later signaling to effector molecules in the Leghorns.

OAS is one of the main IFN effectors that acts in the early phase of viral infection to initiate degradation of viral RNA, resulting in the inhibition of viral replication^33^. After being activated, OAS promotes the synthesis of 2–5 oligoadenylate, which activates RNase L. Upon binding 2′-5′-linked oligoadenylates (2–5 A), activated RNase L degrades cellular and viral RNA^34^. This RNase L-dependent pathway is best characterized; however, recent studies have described OAS antiviral activity through a RNase L-independent pathway^35,36^. In chickens, OAS has been shown to be encoded by only one gene (*OASL*)^37^ and to be upregulated after infection with different viruses^38,39^ including NDV^3,4,21^. The role of *OASL* in the control of NDV replication was confirmed *in vitro*^8^, as the overexpression of *OASL* reduced the replication of NDV and the absence of *OASL* significantly enhanced viral replication. In our study, *OASL* was upregulated by NDV challenge in Leghorns at 2 dpi. Upregulated expression of the *OASL* gene was also observed in challenged Fayoumi and Leghorn in our previous study using RNAseq.^21^. Besides the function described above, OAS also is related to programed cell death^40^. Genetically controlled apoptosis in response to viral infection can help to reduce the spread of progeny virus^41^; however, the role of the OAS/apoptosis interaction in the response to NDV infection is unclear and needs to be further investigated. Lower expression of *OASL* in the spleen of NDV challenged Fayoumis was found at 6 than at 2 dpi. Because the OAS-RNase L pathway requires production of both viral dsRNA and type I IFN together to upregulate OAS and activate RNase L^42^, we suggest the decreased expression of *OASL* at 6 dpi in the Fayoumis is due to the lower type I interferon expression and the lower viral load^20^ in Fayoumis at this time point.

As protein synthesis shut-off in chicken cells can be used as a defense mechanism in response to viral infection^10^, we evaluated the effect of NDV challenge on the expression of genes related to eIF2 family. eIF2 is a protein composed of a regulatory α-subunit (encoded by the *EIF2S1* gene), a tRNA-binding β-subunit (*EIF2S2* gene) and a GDP/GTP-binding γ-subunit (*EIF2S3* gene) that plays a critical role in protein synthesis regulation^43^. The initiation step of translation requires the ternary complex formed by eIF2-GTP and tRNA^Met^ to assemble the pre-initiation complex. To set up the ternary complex, eIF2:GDP binds to eIF2B, a guanine nucleotide exchange factor, which makes the exchange of GDP by GTP^44^. Although the precise mechanism and function of eIF2 subunits has not been fully described in chickens, in mammals this process can be regulated to prevent viral replication and spread to other tissues through protein synthesis shut-off^45^.

PKR is another ISG to be activated after viral infection and is been related to the reduction in protein synthesis^46^. In chicken cells challenged by NDV, PKR undergoes autophosphorylation and phosphorylates the eIF2α subunit^10^. Phosphorylation of eIF2α turns it into an inhibitor of the guanine nucleotide exchange factor eIF2B and prevents the formation of the translation initiation complex eIF2:GTP:tRNA^Met^ that is required for the initiation of protein synthesis^15^. *In vitro* NDV infection caused PKR activation, eIF2α phosphorylation and viral replication inhibition in HeLa cells^18^. Phosphorylation of eIF2α after NDV infection in DF-1 chicken fibroblast cells also induces higher translation of ATF4 and an increase in growth arrest and DNA-damage-inducible protein 34 (GADD34) expression^10^. GADD34 has been described as the link between eIF2α phosphorylation and immune system, however, the mechanism by which GADD34 controls cytokine synthesis after viral infection remains unclear^47^. The phosphorylation of eIF2α can also occur by Protein kinase R-like endoplasmic reticulum kinase (PERK) induced by endoplasmic reticulum stress^16^, however the role of PERK in viral containment in chickens has not been investigated.

Because protein translation is required for viral replication, viruses have developed strategies to avoid protein synthesis shut-off^48^. NDV can manipulate the PKR/eIF2a signaling cascade to favor viral replication by arresting cellular mRNA inside stress granules^49^. We hypothesize that the host cell can use different mechanisms to overcome these viral strategies. Here we show for the first time the relationship of NDV infection on the expression of genes encoding subunits of eIF2 and eIF2B. We observed that NDV challenge downregulated the expression of *EIF2B5* in the Fayoumis at 2 and 6 dpi and in Leghorns at 2 dpi. This suggests that this gene could be downregulated to facilitate protein synthesis shut-off at an early stage of infection.

In mammals, eIF2B contains five subunits. The eIF2ε subunit has the catalytic domain and forms the catalytic subcomplex together with the eIF2γ subunit. They are encoded by *EIF2B5* and *IEF2B3* genes, respectively. The subunits eIF2Bα (encoded by *EIF2B1* gene), β (*EIF2B2* gene) and δ (*EIF2B4* gene) form the regulatory subcomplex^43^. In this study, we assumed that the chicken genes encoding these subunits have the same interactions as in the mammalian model because eIF2 and eIF2B structures have not been fully investigated in chicken. We present the novel finding that infection with NDV could result in the inactivation of the translation initiation factors eIF2 and/or eIF2B in a chicken tissue. We demonstrated that NDV challenged Fayoumis had lower expression than NDV challenged Leghorns of a gene encoding a eIF1B subunit with regulatory (*EIF2B5*) activity, as well as a gene encoding a subunit of eIF2 (*EIF2S3*). Both of these genes could contribute to the difference in relative resistance between these lines. It is important to notice that lower expression of *EIF2S3* gene was also observed in Fayoumis than Leghorns for nonchallenged chickens, which shows a difference between the genetic lines under homeostatic conditions. The activity of eIF2B appears to depend on the phosphorylation of eIF2α as well the phosphorylation in the eIF2B itself ^50^. Other results demonstrate that the catalytic and regulatory subcomplexes in eIF2B can both bind eIF2 independently *in vitro*^51^; the interaction between eIF2 and the regulatory subcomplex is increased by phosphorylation of eIF2, whereas the interaction of catalytic subcomplex and eIF2 it is not dependent on eIF2 phosphorylation. Dysregulation of eIF2Bα may be sufficient to make cells more susceptible to viral infection by neutralizing the consequences of eIF2α phosphorylation^52^. Finally, reduced expression of the eIF2Bε subunit has been related to reduced protein synthesis *in vitro*^53^. All these results show the relation between different eIF2B subunits and the control of protein synthesis. Because inhibition of eIF2B can cause the down-regulation of global protein synthesis by reducing the cellular level of ternary complexes that are available for translation initiation^13^, the differential expression of *EIF2B5* observed between the two challenged lines could be related to the higher ability of Fayoumis to reduce the replication efficiency of NDV. It should be noted that our results were observed in the spleen which could suggest that the eIF2/eIF2B pathway could act as systemic mediators of interferons.

We suggest that the Fayoumis are more resistant to the NDV challenge than the Leghorns due in part to differences in the expression of *eIF2* and *eIF2B* genes, which act on host protein synthesis machinery and, thus, can regulate translation to contain viral replication. Our results describe for the first time the differential expression of eIF2 and eIF2B subunits during NDV challenge in chickens. Because the application of information about resistance mechanisms in disease genetic control studies may lead to better resistance to Newcastle disease, our results suggest that eIF2 family should be the focus of further studies evaluating host genetic resistance to NDV.

## Methods

### Ethics statement

The activities of this experiment were approved by the Iowa State University Institutional Animal Care and Use Committee (IACUC log number 1-13-7490-G). All animal use and procedures were performed according to the approved protocol and in accordance with the relevant guidelines and regulations.

### Animals and experimental design

The effect of NDV challenge on the gene expression in the spleen of two different chicken lines at two- and six-days post infection (dpi) was evaluated. The Fayoumis (M15.2) and the Leghorns (GHs 6), two inbred lines (inbreeding coefficients = 99.95%^54^) were divided in two treatments regarding NDV challenge. At 21 days of age, half of the chickens were inoculated with 200 µl of 10^7^ EID_50_ of La Sota NDV (NDV challenged group, n = 49) through nasal and ocular inoculation routes. The other chickens received 200 µl of phosphate-buffered saline (PBS) (nonchallenged group, n = 40) through same conditions, as described in^19–21^. The chickens from both lines and challenge statuses (NDV challenged or nonchallenged) were euthanized with sodium pentobarbital solution at 2 or 6 dpi. At each dpi, chickens from each treatment were randomly selected for gene expression analysis. Groups are: Fayoumi NDV challenged (n = 4), Fayoumi nonchallenged (n = 3), Leghorn NDV challenged (n = 4) and Leghorn nonchallenged (n = 3). Samples were collected from spleen and placed into RNAlater solution (ThermoFisher Scientifc, Waltham, MA) and stored in a −80 °C freezer until the RNA isolation procedure. The samples used for gene expression analysis in the current study were previously used by^21^. The detailed description for the experimental design and animal activities can be found in^20^.

### Gene expression

The spleen tissues were homogenized using mechanical disruption and total RNA was isolated using the Ambion RNAqueous Total RNA Isolation Kit (Thermo Fisher Scientific, Waltham, MA) according to the manufacturer’s protocol. All the samples were treated with DNase using the DNA-free kit (Thermo Fisher Scientific, Waltham, MA) according to the manufacturer’s recommendations. Proper quantity and quality of the RNA samples were ensured respectively through assessment by NanoDrop ND-1000 UV-vis spectrophotometer (Thermo Fisher Scientific, Waltham, MA) and the RNA 6000 Nano kit on the Agilent 2100 Bioanalyzer (Agilent Technology, Santa Clara, CA). The RNA integrity numbers are average = 8.2, median = 8.4, range = 4.1–9.5.

Gene expression was evaluated through One-Step qPCR using the QuantiTect SYBR® Green RT-PCR Kit (Qiagen, Germantown, MD). The amplification reaction consisted of 1 µl of RNA at 50 ng/µl, 1 µl of each primer (forward and reverse) at 15 pM, 12.5 µl of QuantiTect® SYBR® GREEN RT-PCR master mix, 0.5 µl of QuantiTect® RT mix and water to a total volume of 25 µl. The thermal cycling parameters for all genes were as follows: incubation at 50 °C for 30 min, hot-start at 95 °C for 15 min, followed by 40 cycles of denaturation at 94 °C for 15 s, annealing at 60 °C for 30 s, and extension at 72 °C for 30 s, and ending with a melt curve from 65 °C to 95 °C. Chicken specific primers (Table 2) used for the amplification reactions were designed based on the gene sequences deposited at www.ncbi.nlm.nih.gov (*Gallus gallus* Annotation Release 104) using the Primer Blast Tool. *IFNA* is a multi-copy gene family, and since primers used for *IFNA* in this study will bind to more than one of these gene copies, *IFNA* expression should be considered as representing the gene family. Primers sequences for the housekeeping gene (28 s gene) were previously reported^55^. All of the reactions were performed in triplicate and each plate contained both a negative and a no reverse transcriptase control.

**Table 2:** Primer sequences used for quantitative real-time PCR.

Gene	Sequence (5′-3′)		NCBI access number/ Reference
	Forward primer	Reverse primer	
*TLR-7*	CGGAAAATGGTACATCATGC	AAAGTTTTGGGAAACCAACG	See reference^56^
*IFNAR1*	TCAGGTTCGAAAAATGTGGCT	GGTAGTCTCTGGAGCAAGATCA	XM_015299270.2
*IFNA*	GACAGCCAACGCCAAAGC	GTCGCTGCTGTCCAAGCATT	See reference^57^
*IFNB*	CTGGATTGACCGCACACGCCA	GGGAGCGCGTGCCTTGGTTTA	See reference^57^
*IL1B*	GCTCTACATGTCGTGTGTGATGAG	TGTCGATGTCCCGCATGA	See reference^58^
*IL6*	GCT CGC CGG CTT CGA	GGTAGGTCTGAAAGGCGAACAG	See reference^58^
*TNFA*	CGCTCAGAACGACGTCAA	GTCGTCCACACCAACGAG	See reference^59^
*JAK1*	AGAGGCTGAGGGGTACGG	ATCTTCACGCTCTCCAAGGG	XM_015290965.2
*STAT1*	CGTCCGTGCGGGTATTTCTG	AGCTGGTGAACTTGCTCCAA	XM_025152161.1
*ATF4*	GTTCTCCAGCGACAAGGCTAA	CTCCATGCCAGAGAAGGCATC	NM_204880.2
*OASL*	GTCGACATCCTGCCTGCTTAC	GAAGCTGGGGGAGAAATCGC	XM_015293006.2
*EIF2AK2*	CGTCGACGTGGACATGAGAG	GCTGCAGCTTTTGCTTCCTT	XM_015283611.2
*EIF2AK3*	GTGGAGGACGATGTGACCG	AGGATCCAGGGCAGCAATTC	XM_420868.6
*EIF2S1*	GACGTCTGACTCCACAAGCA	GTGGAACAGTTCAAGCCTGC	XM_025150723.1
*EIF2S2*	CCTGGCATTTTTGTTGGCAGA	CCGACACGTATGACAGGTGA	NM_204597.1
*EIF2S3*	CCGACCCGAATGTTACCGAT	TCGTGACCAGGACAATCCAC	NM_001006260.2
*EIF2B1*	CAGGACCGAGGAGAGACCAT	GTATTCCAGCGAGGTGAGGC	XM_003642192.4
*EIF2B2*	CTCCACGCCGCTCATTGTAT	AGTCAAACACAGGGCAGTGAA	XM_015287388.2
*EIF2B3*	TGATCGGAAGTGATCAGAGGC	CTGTTCAGTGCCCACAATGAC	XM_015291004.2
*EIF2B4*	CGCAGCCCCGCGTTA	AGCTCCGCTTTGCTTTTGC	XM_423512.6
*EIF2B5*	GAGAAGCAGAGGAGAGGGGA	CATGGCCACATTTGCCATAGG	XM_015291568.2

*TLR7*, *toll*-*like receptors 7; IFNAR1*, IFN-α receptor subunit 1; *IFNA*, interferon *α* multi-copy gene family*; IFNB*, interferon β*; IL1B*, interleukin 1β*; IL6*, interleukin *6; TNFA, tumor necrosis factor α; JAK1*, janus kinase 1; *STAT1*, signal transducer and activator of transcription 1; *ATF4*, activating transcription factor 4; *OASL*, 2′-5′-oligoadenylate synthetase like; *EIF2AK2*, eukaryotic translation initiation factor 2 alpha kinase 2; *EIF2AK3*, eukaryotic translation initiation factor 2 alpha kinase 3; *EIF2S1*, eukaryotic translation initiation factor 2 subunit alpha; *EIF2S2*, eukaryotic translation initiation factor 2 subunit beta; *EIF2S3*, eukaryotic translation initiation factor 2 subunit gamma; *EIF2B1*, eukaryotic translation initiation factor 2B subunit alpha; *EIF2B2*, eukaryotic translation initiation factor 2B subunit beta; *EIF2B3*, eukaryotic translation initiation factor 2B subunit gamma; *EIF2B4*, eukaryotic translation initiation factor 2B subunit delta; *EIF2B5*, eukaryotic translation initiation factor 2B subunit epsilon.

### Data analysis

Gene expression data were evaluated as the adjusted Ct (cycle threshold) value using the following formula: 40 − [(mean test gene Ct) + (median 28S Ct − mean 28S Ct) × (test gene slope/28S slope)]. Slopes were determined with five points of a 10-fold dilution series using pooled RNA for the housekeeping gene and target-specific amplicons as the template to determine PCR efficiency of each primer/gene set. Median 28S Ct represents the median Ct value of all individual samples for the housekeeping gene. Additionally, the fold change was used to display the differential gene expression in Figs. 1–3.

The expression of each gene (expressed as adjusted Ct values) was fit to a linear model (adjusted Ct ~ group, where group incorporates line, challenge status and dpi) using R 3.6.1 and analyzed by ANOVA using the car package 3.0–2. The multcomp package 1.4–10 was used to test pairwise contrasts between groups, testing the effect of line, challenge status, and dpi (12 total comparisons) and included False Discovery Rate (FDR) correction based on the Benjamini-Hochberg procedure. FDR-adjusted p-values were considered significant at *FDR* < 0.05; *FDR* < 0.1 was used to show results of genes that had an expression pattern similar to the others in a pathway. All FDR-adjusted p-values for each gene from all 12 contrasts are reported in Supplementary Table S1.

## Supplementary information

Supplementary Table S1.

## Data Availability

The datasets generated during the current study are available from the corresponding author on reasonable request.
